# Winter Bathing in Denmark: A Qualitative Case Study on Winter Bathing’s Impact on Mental Health

**DOI:** 10.3390/healthcare12202076

**Published:** 2024-10-18

**Authors:** Elisabeth Bomholt Østergaard, Anders Aagaard Petersen, Linda van den Hengel, Anne Møller Jensen, Nikolaj Bonde Jensen, Pernille Wobeser Sparre, Jesper Dahlgaard

**Affiliations:** 1 Research Programme for Mind and Body in Mental Health, Research Centre for Rehabilitation, VIA University College of Aarhus, 8200 Aarhus, Denmark; pspa@via.dk (P.W.S.); jesd@via.dk (J.D.); 2 Day Treatment Centre South/Kolt, Kunnerupvej 196, 8361 Kolt, Denmark; andersaagaaard@live.dk; 3 Anthropology of Pedagogic, Aarhus University, 8000 Aarhus, Denmark; 4 Department of Physiotherapy, VIA University College of Aarhus, 8200 Aarhus, Denmark; linda.vdh@live.dk (L.v.d.H.); nbonde8@gmail.com (N.B.J.); 5 Social Work, Aalborg University, 9220 Aalborg, Denmark; annemoellerjensen@hotmail.com; 6 Blue Spirit Surf School, Ndr. Strandvej 42, 8400 Ebeltoft, Denmark

**Keywords:** winter bathing, winter swimming, blue nature, blue space, ice-cold shock, mental health, mental peace, sense of safety, social connectedness, anchor to the present moment, normal bodies, relaxed attitude to one’s own body

## Abstract

Background: Mental health disease constitutes 25% of the total burden of disease in Denmark. In the last few years, an increasing tendency for seeking the ocean has arisen, and winter bathing is one of the outdoor activities with a large rise in popularity. Objectives: The purpose of this study was to explore if and how regular winter bathing in blue spaces can affect people’s mental health. Methods: This study employed a qualitative design using fieldwork with semi-structured interviews with four members from three different winter bathing clubs. The empirical data were thematically analyzed followed by theoretical analysis. Results: The results revealed five themes: Social Connectedness; Blue Nature as a Free Space; The Motivation Behind Winter Bathing; It is More Than an Ice-Cold Shock; and Finding Mental Peace in Winter Bathing. The results suggest that winter bathing in blue spaces contributed to improved mental health for the informants. Conclusions: In conclusion, regular winter bathing enhanced the participants’ mental health. A relatively short time in the ice-cold water provided mental peace which transferred to the rest of daily life in general, which emphasizes the mental health-promoting potential of winter bathing. Positive emotions were cultivated through exposure to blue spaces, including inner calm and a sense of safety. Winter bathing also served as a space for social connectedness and community building, facilitated by the club environment in which meaningful social interactions occurred.

## 1. Introduction

Globally, mental health disease is one of the 10 leading causes of disability, and depression is the leading cause of years lost due to disability, projected to be ranked the number one global burden of disease by 2030 [1]. In Denmark, mental health has decreased from 2010 to 2023 for all age groups and especially for the youngest groups aged 16–24 and 25–34 [2,3], and mental health disease is now the most prevalent disease constituting 25% of the total burden of disease [4].

Mental health disease increases the risk for the development of physical diseases, especially heart and vascular disorders [2], and even a low level of mental health disease increases the risk of early death [5]. The costs are enormous for the individual as well as socioeconomically due to increased contact with the healthcare system, increased unemployment, and lower levels of education [6]. The total amount of healthcare expenditures in 2022 in Denmark was DKK 265.7 billion corresponding to about EUR 1992.8 [7].

Therefore, mental health has been a political focus these years and the Danish government has agreed to strengthen and promote the Danes’ mental health—especially in the area of psychiatry—for the next 10 years [8]. However, there is a need for research and development of preventive strategies and initiatives that can reverse the negative downward spiral in mental health thereby preventing the risk of developing mental health disease and associated physical co-morbidity [4]. Thus, novel approaches must be sought for mental health promotion and prevention of mental health disease, with the potential to reverse the downward spiral in mental health and reduce the socioeconomic costs.

A growing body of evidence emphasizes the connection between different blue nature activities and increased mental health and psycho-social well-being [9,10,11,12,13,14,15,16,17,18]. Being near, in, under, or on the water can make people happier, healthier, more connected, and better at what they do [19].

Through history, humans have always been drawn to the sea, lakes, and rivers to live, relax, be active, and achieve healing, and through the recent five to ten years, more and more Danes have been drawn to blue nature and blue spaces participating in different forms of outdoors activities near, in, and on these aquatic environments [20].

Denmark is a small country with 5903 mio. inhabitants, but with more than 7000 km seaside and many lakes and rivers; it has many aquatic environments and can be characterized as a blue country [20]. Danes are increasingly interested in blue nature and blue space activities. In 2018–2019, the athletic association obtained 3000 new members of the six blue space activities: canoe and kayak; rowing; yachting; water ski; surf and raft and stand-up paddling; and open-water swimming [21]. Canoe and kayak and surf and raft and stand-up paddling have had a massive increase in number of members [21], and winter bathing is also one of the blue nature activities with a massive increase in members. In 2022, more than 70,000 people in Denmark were members of a winter bathing club [22]. Thus, winter bathing is embedded in the Danish culture. Intriguingly, Finland has been rated as having the happiest people in the world year by year for the last seven years [23], and winter bathing is deeply embedded in the Finnish culture; therefore, there may be a connection.

Our research asks the following question: “Can winter bathing be a feasible approach for mental health promotion and prevention of mental health disease?”.

Few studies have shown various health benefits of winter bathing such as increased fat burning, reduced chronic inflammation, and a positive effect on stress regulation [24], improved sleep and well-being in people with depression [25], and improved pain relief and general well-being [26,27].

However, to assess whether winter bathing may be a feasible approach for mental health promotion and the prevention of mental health disease, there is an empirical gap and a need for further research, especially for qualitative studies, to gain insight into people’s experiences with winter bathing including its long-term impact on mental health [1,24,27].

The aims of this study were to explore the experiences from healthy young adults engaging in regular winter bathing outdoors in blue nature for at least one year and how this could affect mental health. Furthermore, the aim was to create relevant knowledge for health professionals to meet the need for novel approaches that potentially could be suggested for mental health promotion and prevention of mental health disease, improving well-being and decreasing associated socioeconomic costs.

### 1.1. Definitions

Winter bathing was defined as bathing in the sea or lake, when the water temperature is 10–15 degrees or lower. In Denmark, this typically occurs in the wintertime from October to April [28]. In our study, winter bathing included sauna accessibility to generate warmth in the body between each dip in the sea, and it also included swimming or treading in cold water, cold-water dipping, and standing in cold water.

This study uses the WHO’s definition of mental health as a state of mental well-being that enables people to cope with the stresses of life, realize their abilities, learn well and work well, and contribute to their communities [29].

**Figure 1:**
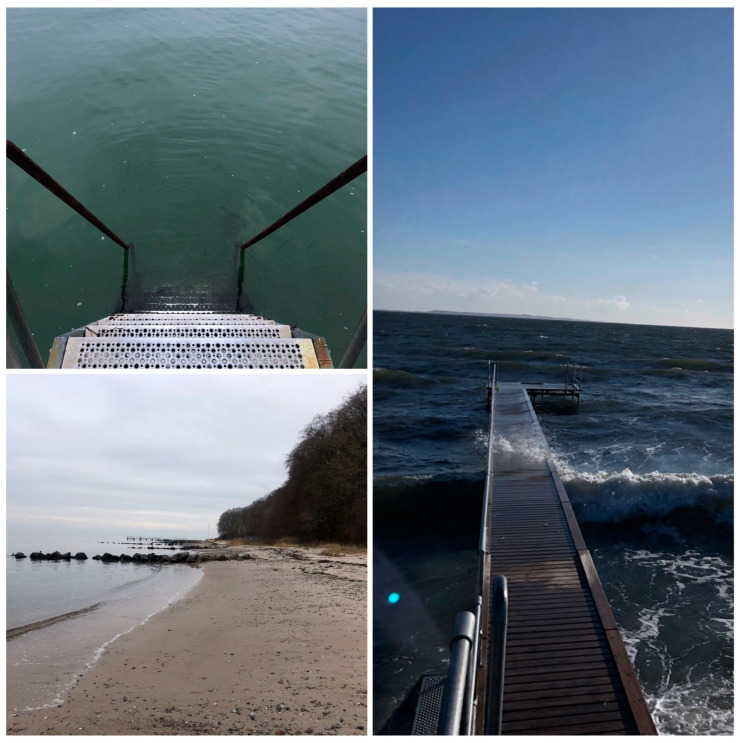
Blue nature. Photo: Linda van den Hengel.

Blue spaces are often defined as water bodies and all forms of natural and manmade surface water [30,31] and in order to include the surroundings of blues spaces, we chose to use the notion blue nature in this project. Blue nature is defined as areas by, near, in, on, and under water. In this study, we focused on areas by, near, in, and under water. Examples of blue nature are illustrated in Figure 1.

Health prevention was defined as health-related activities intended to prevent the occurrence and development of diseases, psycho-social problems, or accidents, thereby promoting public health [32].

The concept of health promotion was defined as health-related activities, frameworks, and processes enhancing individual health and public health by creating frameworks and opportunities for the individual, thus promoting the individual’s resources and skills for action [32].

The social aspect encompasses encounters between people seeking connection and companionship and interactions between individuals like engaging in conversations, winter bathing together, or reflecting upon one another.

### 1.2. Theoretical Perspectives

Our theoretical perspectives were based on the concepts of sense of coherence by Aaron Antonovsky [33], the phenomenology of perception by Maurice Merleau-Ponty [34], self-efficacy by Albert Bandura [35,36,37], the self-determination theory by Deci and Ryan [38,39,40,41], body image by Bob Price [42] and biophilia by Edward O. Wilson and Stephen R. Kellert [43,44].

According to Antonovsky, three important factors for being able to manage the experiences and challenges through life, for having a sense of coherence, and for developing and maintaining well-being are comprehensibility, manageability, and meaningfulness [33]. Comprehensibility is primarily the cognitive aspect about understanding challenges through life, and when challenges are experienced predictably and with some structure, comprehensibility increases. Manageability is the behavioral aspect about the individual’s resources, whether inner resources or support from others, and the ability to manage challenges through life. Strong manageability enables the individual to manage many challenges, to cope with and solve problems, and to see them as challenges rather than burdens.

The phenomenology of perception by Merleau-Ponty contributes to a holistic understanding of the human being in which the individual should be understood as a whole, body and mind inseparable, with the body being an anchor to the present moment [34,45,46]. According to this concept, ‘I am my body’; body and mind are ‘one thing’; there are no two things to separate; they cannot be separated from each other; and body and mind will influence each other, and in other words, the effect can yield either result.

Self-efficacy by Albert Bandura concerns individuals’ beliefs in their own abilities, and self-efficacy can be fostered through experiences with role models [35,36,37]. This theory emphasizes the importance of support from others and their acting as role models. Support and recognition from social surroundings are important factors for feeling safe and achieving desired goals. People learn through observing, imitating, and modeling others’ behavior. Other people can serve as role models, and observing others carrying out the activity successfully can contribute to strengthen one’s self-efficacy.

Self-determination theory by Deci and Ryan is among others regarding three fundamental needs: autonomy, relatedness, and competence, and all three are important for our self-determination and well-being [38,39,40,41]. Autonomy is about living and managing one’s own life. Relatedness is about feeling connected to other people and being a part of a community, and relatedness and connectedness can contribute to intrinsic motivation. Competence is about the experience of success, creating meaningful results, and coping with tasks. These fundamental needs are essential and necessary for psychological growth, integrity, and well-being, and the more they are satisfied, the healthier the individual can be [38].

Body image theory by Bob Price describes how humans through childhood and adulthood form a mental image of the body through various activities and experiences [42]. Three elements, body reality, body presentation, and body ideal, all together influence body image. Body reality is about the physical appearance, e.g., height, being slim, muscular, etc. Body presentation is about showing the body through nonverbal communication and dressing. Body ideal is about one’s own view of the ideal body.

The biophilia theories by Wilson and Kellert concern humans’ connectedness to nature and explain how a sense of belonging to nature is genetically determined, and how sensory experiences in nature can evoke emotional and physiological responses [43,44]. Nature can bring about memories from and connection to one’s childhood and upbringing as a basis for developing positive feeling and for feeling secure, and the term philia refers to safe and secure environments where positive emotions can develop and unfold.

## 2. Materials and Methods

### 2.1. Design

This study employed a qualitative case study design consisting of fieldwork with semi-structured interviews conducted in November and December 2023 in Denmark. The reason for using a qualitative research design was to explore the participants’ experiences with winter bathing. The reason for using a case study design was to obtain an in-depth exploration and analysis of individual experiences. The first interview was obtained indoors at VIA University College and the following three interviews were obtained outdoors in blue nature walking along the seaside and sitting at the seaside in order to create optimal conditions for the participants to recall their experiences and to reflect on them. The interview guide is available as shown in Appendix A.

### 2.2. Preunderstanding

By explicating our preunderstanding, we increased the ability to release ourselves from preunderstanding [47,48]. We liberated ourselves from preconceived notions or preunderstanding by clarifying them and writing them down. All authors were familiar with the context of participating in winter bathing and our preunderstanding is influenced by this, such as the sound of the waves breaking on the shore and the refreshing wind against the face, or experiencing an adrenaline rush and a cold shock in the water, while the skin tingles and breathing changes. The authors have personally experienced how circulation increases after each dip in the sea, which generates warmth in the body, followed by a sense of mental calm and well-being. We have used winter bathing as a sanctuary in a busy everyday life, while some of us have also used it as a tool to get through difficult periods in life. The interest in this subject originates in a shared fascination with blue nature. We have all lived close to the coast and have been engaged in various water activities, providing a basis for exploring this from a health professional perspective. This made us each a sort of ‘native’ anthropologist, and it is sometimes questioned if it is possible for a native anthropologist to create a necessary distance to the participants and the field, but on the other hand, it is also assumed that the native anthropologist is able to contribute with more authentic knowledge than the non-native anthropologist [49].

Our preunderstanding was also influenced by the interviewing authors being physiotherapists. This means having a bio-psycho-social-existential approach to peoples’ health and a perspective of the body as being an important entry point to increase mental health and health promotion in general.

### 2.3. Entering the Field

Two winter bathing clubs did not want to participate and refused to ask their members to participate. We then recruited participants through our contacts within the winter bathing community in Aarhus. These contacts directed us to potential participants with a regular practice of winter bathing. We made agreements with five participants, but unfortunately, one of them became sick and cancelled the appointment.

### 2.4. Participants

Four participants aged 25–32 (two male and two female) from three different winter bathing clubs participated in the semi-structured interviews. They had all participated in winter bathing regularly 1–3 times a week for a minimum of one year and up to three years. As inclusion criteria, the participants should have felt healthy and have participated in winter bathing for a minimum of one season, that is, one year.

P1: 25-year-old healthy man, no remarks about mental health, bachelor’s degree student, grew up in the country, and moved to a larger city in Denmark to study.

P2: 27-year-old healthy woman, no remarks about mental health besides a period with stress two years ago, bachelor’s degree, full-time work in the healthcare field, grew up in the country, and moved to a larger city in Denmark to study, now living there.

P3: 32-year-old healthy woman, no remarks about mental health, master’s degree, job seeker, grew up in the city, and moved to a larger city in Denmark to study, now living there.

P4: 25-year-old healthy man, no remarks about mental health, master’s degree student, grew up in a little village in the country, and moved to a larger city in Denmark to study.

### 2.5. Ethics

This study was conducted in accordance with the Declaration of Helsinki [50]. The design, aim, and procedures were evaluated and approved by the Central Denmark Region Committee on Health Research Ethics, Skottenborg 26, DK-8800 Viborg, Denmark (record number: 1-10-72-109-23).

Prior to the interview, the participants were thoroughly informed of the study’s objectives, publication of results, and the possibility of withdrawal from this study. Written informed consent was obtained from all participants to participate and to publish this paper.

We anonymized the data and names of the participants to protect individual confidentiality [50,51]. The participants’ names were anonymized with the following pseudonyms: P1, P2, P3, and P4. Audio files and all written material were kept safe and secret behind passwords only known by the authors.

The researcher’s position potentially influences the participants’ answers in different ways whether the researcher, e.g., is a psychologist, doctor, social worker, or physiotherapist, and it potentially influences the researcher’s interest and with it questions as well because of the different areas of expertise. For ethical reasons, authors 2, 3, 4, 5 introduced themselves as physiotherapists, but they emphasized that they were very interested in both mental, social, and physical experiences. There were no affiliations/belongings between the participants and the researchers. This research received no external funding.

### 2.6. Data Collection

Semi-structured interviews were recorded with an iPhone, audio only, and were transcribed in full length including pauses and repetitions, and finally, they were reviewed again simultaneously with listening to the recordings to ensure reproduction and preservation of the original meanings [52]. During transcription, persons and places were blurred to protect anonymity [50,52]. The transcription process led to new reflections and perspectives supplying the basis of the data analysis [47,48].

### 2.7. Thematic Analysis of the Empirical Data

Our perspective was phenomenological, temporarily setting aside preconceived notions, and the empirical data from four interviews were thematically analyzed following Malterud’s four-step approach based on Giorgi’s psychological phenomenological thematic analysis [47,48].

In the first step, we thoroughly reviewed the transcribed empirical material, formed initial impressions, and identified preliminary themes. We were very conscious about our preunderstanding with the aim of not allowing it to influence us. We identified 14 preliminary themes and gathered them in five groups:

1. Group 1: Social, body image;
2. Group 2: Nature;
3. Group 3: Motivation, prioritization;
4. Group 4: Bodily experience, the importance of the sauna, health benefits, bodily experience, structure, practical experience, routine;
5. Group 5: Peace in my mind, the experience with winter bathing.

In the second step, we identified empirical data for each theme.

At step three, we condensed the empirical data for each theme to obtain the essence of each theme. We expressed the essences by synthetic quotations consisting of several compiled quotations and observations with equal essence. During this process, we selected so-called golden quotations [47,48] which were kept in their original form.

At step four, we re-contextualized each theme for an academic context. The final themes, which are also headings in the Results section, are (1) Social Connectedness, (2) Blue Nature as a Free Space, (3) The Motivation Behind Winter Bathing, (4) It is More Than an Ice-Cold Shock, and (5) Finding Mental Peace in Winter Bathing. Subsequently, we analyzed the empirical data with theoretical perspectives.

### 2.8. Analytical Perspectives

We were very conscious about the balance between the empirical material and theory; of not allowing the theoretical approach to overrule the empirical material; and above all, of allowing the empirical material to step forward without too much theory as, for example, underlined by Wolcott and Bundgaard [53,54].

Therefore, at first, we analyzed the empirical data, and *after* the empirical analysis, we discussed our results with the theoretical perspectives of Antonovsky [33], Merleau-Ponty [34], Bandura [35,36], Deci and Ryan [38,39,40,41], Bob Price [42], and Wilson and Kellert [43,44], as introduced in the introduction section.

## 3. Results and Analysis

### 3.1. Themes

Five main themes were identified from the data: (1) Social Connectedness, (2) Blue Nature as a Free Space, (3) The Motivation Behind Winter Bathing, (4) It is More Than an Ice-Cold Shock, and (5) Finding Mental Peace in Winter Bathing.

#### 3.1.1. Theme 1: Social Connectedness

Being a member of a winter bathing club created a physical and social setting where the members accommodated each other’s needs and had a mutual respect for each other’s differences. A participant described how the sauna became an arena where spontaneous conversations arose and if needed, space was given to self-focus:

*“There is a culture in the club where everyone greets each other. We want to talk and engage with each other. But it is also respected if someone just needs to be alone for a bit. (…) One feels welcomed”.*

(P2)

The participant described how members shared and cultivated something special in the club—something unique to them. A bond of trust was formed among the members, with unwritten rules about how they interacted. Room was made for people’s differences and individual needs. Differences thrived, which made the winter bathing clubs a gathering place for a wide range of citizens. This large diversity of people positively exposed one participant to more realistic and ordinary bodies in real life, which was not comparable to the participant’s previous perception. The participant shared the following:

*“It matters that you must be naked. The thing about seeing other bodies, completely normal bodies, not mannequins, models, fitness trained and muscular people etc. I experienced that as really healthy, and you get a more realistic view of what is normal. It has contributed to giving me a more relaxed relationship to the body. (…) One is not better than others, and there is a place for everyone”.*

(P3)

*“When I was in my twenties, I was very concerned about my appearance, my body and what I was eating. I got much more relaxed about this when I started participating in winter bathing. I saw that everyone looks different. Nothing is better than anything else, and there is room for everyone. All this has a calming and pleasant effect on me. It has been a good and healthy experience for me”.*

(P3)

*“Though you know that people do not look like the models, it makes a great difference to see it (…). It is such wonderful experiences, that would not have been a part of my everyday life if it had not been for the winter bathing”.*

(P3)

The participants were exposed to authentic bodies and experienced a social openness and acceptance in the club, leading to a changed body image, which had been distorted in the past. This aligns with Bob Price’s theory of body image describing how humans form a mental image of the body through various activities and experiences [42].

The quotes above suggest that the participants’ body images were altered through exposure to real bodies in the winter bathing club across all three components: body reality, body presentation, and body ideal.

Another participant described how being a member of a winter bathing club facilitated situations where social interactions with other members occurred spontaneously and could form a foundation for a deep and meaningful conversation, as the following quote described:

*“I was sitting in the sauna and talking to someone, who said that winter bathing really had an impact on her life and has contributed with many positive things. I found that a bit amusing. I think, I had kept it to myself a bit, because it can be difficult to explain. We clearly recognized what each other was saying. It was pretty cool to experience.”*

(P2)

This example showed how the social setting and the mutual bond formed in the winter bathing club acted as a foundation for a spontaneous conversation between the participant and another member, which evolved into a personal dialogue about challenging times in life and their unique connection through winter bathing. In a perspective from Deci and Ryan’s self-determination theory about the fundamental needs autonomy, relatedness, and competence [38,39,40,41], the participant experienced relatedness. Relatedness refers to feeling connected to other people and being a part of a community. Relatedness is instrumental for experiencing belongingness and the development of close relationships. Both constitute antidotes to loneliness, and thus may support not only mental well-being, but also the sense of motivation, in addition to mental, physical, and social dimensions of health [38,39,40,41]. Loneliness increases the risk of early death much more than other risks as, for example, obesity and physical inactivity. The influence of social relationships mortality risk is comparable with well-established risk factors for mortality as, for example, obesity and physical inactivity, and social relationship–based interventions represent a major opportunity to enhance not only the quality of life but also survival [55].

The participant experienced this connection with another member as they were able to recognize each other’s experiences. This established the basis for sharing personal matters, which may have been difficult before in other settings of life. Even though this interaction was brief, a close relationship formed between the participant and the other member in the sauna, characterized by mutual understanding, confidentiality, and a sense of belonging.

#### 3.1.2. Theme 2: Blue Nature as a Free Space

The participants described the ocean areas with abundant impressions under the influence of wind and weather. The sight of the horizon, the scents, and the sound provided the framework for the wildness of nature, experienced by the participants as having a therapeutic effect that provided them with inner peace. All participants lived in cities, but they still considered coastal areas as unspoiled sanctuaries, as supported by the following quote:

*“I think the ocean offers a different calmness than the forest. There is something very therapeutic about the ocean with the waves, for me. I take many walks by the ocean, (…) and it’s if as the ocean is more unspoiled. It’s wilder”.*

(P4)

The areas around the ocean provided the participants with the opportunity to experience nourishment and new energy within themselves and provided a sense of being in untouched nature, even with the vibrant city life in the background. All participants mentioned that they associated the ocean with a recognizable familiarity from their upbringing, providing them with inner peace. This was expressed in the following quote:

*“I’m from the countryside as well, so I grew up with the ocean around me and have always used nature a lot. For me, it’s also a bit like home. It’s quite important for me to live close to the ocean”.*

(P2)

This participant expressed how she experienced a sense of security in the ocean areas that could be traced back to her upbringing, leading to continuing to make use of embracing and feeling significantly connected to the ocean. E. O. Wilson explains with the biophilia theory [43,44] that a sense of belonging to nature is genetically determined, where sensory experiences can evoke emotional and physiological responses. Philia is a term for a secure environment where positive emotions can develop and unfold. This was experienced by all participants.

#### 3.1.3. Theme 3: The Motivation Behind Winter Bathing

One may wonder why some choose to swim in ice-cold water during the winter. We investigated this and the motivation behind winter bathing with perspectives from Bandura’s theory of self-efficacy [35,36,37] and Deci and Ryan’s self-determination theory [38,39,41].

Three of the participants emphasized that winter bathing was something they gave high priority to in their daily lives. There were various reasons for this, but common to them was that the motivation largely came from within. It was pure desire that propelled them to plunging into the ice-cold seawater, because the activity was enjoyable in itself.

Three of the participants were intrinsically motivated and therefore, according to self-determination theory, their behavior was in line with one of the strongest and most enduring motivational types [38,39,41].

The social aspect of winter bathing was significant and served as a driving force, as individuals could engage in the activity collectively with others. One participant pointed out that the reason she began winter bathing was because she could go together with a friend. Furthermore, she knew several of the other members of the club, which also contributed to her own initiation, as expressed in the following quote:

*“I’m not sure, I would have started if there hadn’t been anyone I knew”.*

(P3)

The social aspect, emphasized in the above quote, provided an impression of the club offering a sense of belonging and community. According to self-determination theory, it is highlighted that social relationships enhance intrinsic motivation [38,39,40,41]. According to Bandura, self-efficacy can be fostered through experiences with role models [35,36,37], which could explain why three of the participants started winter bathing. If friends had not persuaded them and offered to take them along, fewer of the participants might have taken their first plunge into the cold water.

Another participant brought someone along to demonstrate how skilled she was at enduring staying in the ice-cold water for a long time. This gave the impression that winter bathing provided her with something social, beside development and mastery of the activity. She became better at staying in the ice-cold water over time. These experiences of mastery, managing, and the sense of competence ensured the desire to engage in winter bathing [35,36,37,38,39,40,41].

All participants focused on improvement. One wanted to toughen his body, and others aimed to build tolerance and enhance themselves from week to week. A participant pointed out that he used winter bathing to optimize performance in other arenas of life because it had many health benefits, which was one of the reasons for him to be engaged, which is evident from the following quote:

*“It should be incredibly healthy”.*

(P1)

The participants also craved peace and tranquility, and they emphasized that they achieved peace and tranquility during winter bathing. For example, this could be achieved by visiting the winter bathing club at off-peak hours when the sauna was not overcrowded, and it could be achieved by lowering their ears under the ice-cold water to experience silence. The sauna was also an essential element of bathing for three of the participants. It made bathing easier, as there was no need to bring a lot of warm clothing, and it resulted in multiple dips. Some of the participants expressed the following about the sauna:

*“It’s a deal breaker, if there wasn’t a sauna…”.*

(P4)

*“I think, I joined a club, because of the sauna. Jumping into the water, you can do that anywhere”.*

(P1)

#### 3.1.4. Theme 4: It is More Than an Ice-Cold Shock

When control of the breathing was achieved, and the ice-cold water was resisted, a calmness, almost a meditative state, was experienced, as highlighted by the participants. We explored those experience with the theory of the sense of coherence (SOC), which comprises the following three components: comprehensibility, manageability, and meaningfulness [33], originating from the Israeli sociologist Aaron Antonovsky.

All the participants had a fixed routine they followed each time. It was important for them to know in advance how many times they needed to go into the water and the sauna, providing them with predictability and structure. The following quote describes the importance of the routine:

*“I have a fixed routine every time. Firstly, I go into the water, followed by the sauna, and I repeat that three times”.*

(P2)

The quote above is an example showing that predictability was important for the participants in their winter bathing. It created a greater degree of comprehensibility for the participants, as they were familiar with the duration and extent of the activity, aligning with the SOC [33]. However, it was not only the routine that was crucial for the participants, but also the mental preparation before the dip into the icy water, as described in the following quote:

*“When I go down the stairs into the water, I always try to prepare myself: now it’s going to be cold”.*

(P3)

The mental preparation helped by equipping the participant for the ice-cold shock and created predictability, demonstrating a high degree of comprehensibility. The participant was aware of what the activity entailed and was mentally prepared for the physical discomfort that the cold water could cause. Breathing was used as an active part of winter bathing. A participant viewed breathing as an element which he/she could train in order to keep the physical response/discomfort of the cold water at bay. It was an important element in winter bathing, as described in the following quote:

*“Actually, I think that throughout the last season, the primary focus has been to learn to stay in the cold water and to be able to control my breathing. Because often I was only in the water for maybe 5 s, before I went up and into the sauna”.*

(P4)

The cold water could become uncomfortably intense for the participants, and after some time could cause a painful bodily sensation. Breathing was then a resource to withstand the significant strain and the temptation to step out of the cold water, which reflected a high degree of manageability [33]. The ability to endure the discomfort with the awareness that it was temporary indicated a high degree of manageability. This was articulated by a participant:

*“For me, it’s about getting over that barrier of about 20 s. (…) Then I know it gets better after the initial shock has subsided”.*

(P1)

Even though the water was ice-cold, the participants were aware of the positive effects it had on them both in the water and afterwards. They forgot the challenges of life and were simply present in the moment. A meditative space for solely being present in the moment and for racing thoughts put on hold for a while was created. The following quote described the reward for plunging into the cold water and enduring the physical discomfort:

*“I quickly began to feel comfortable, especially getting my ears underwater, and the silence it brings is really pleasant for me. (…) I can feel completely cut off from the outside world in a good way”.*

(P3)

This quote left the impression that even though the water was cold and not very inviting, a greater sense of meaning was achieved through winter bathing. It motivated the participant to invest further time in the activity, as it created silence and a feeling of being away from the outside world.

The experienced meaningfulness did not occur solely in the water, but also throughout the remainder of the day. Winter bathing created a calmness in the body and mind, which the participants carried with them for the rest of the day, providing a sense of having done something beneficial for their bodies and for themselves. The following quote described the subsequent effects arising after winter bathing:

*“It can give me calmness, either becoming very tired afterwards, or becoming very refreshed afterwards, depending on whether I finish in the water or in the sauna”.*

(P2)

This participant described how winter bathing could be used in different contexts, depending on the desired outcome. It could be either refreshing or calming.

#### 3.1.5. Theme 5: Finding Mental Peace in Winter Bathing

The participants used winter bathing as a break from the stressors of everyday life and a break from their thoughts. They experienced the cold shock against their bodies which triggered a mental break in their brains. One of the participants described the meditative aspect of winter bathing and how daily life faded into the background with the following quote:

*“It can be quite meditative to sit there in the water. You quickly forget other things and have a strong focus on being present in the moment, because it’s challenging to think about other stuff when you’re getting hit with that cold water constantly. So, I don’t think much is happening in your head”.*

(P1)

The quote above indicated how the participant experienced a mental break and could be more present in the moment. According to the perspectives of Merleau-Ponty, the individual should be understood as a whole, body and mind inseparable, with the body as the anchor to the present [46]. By being fully present in the body in the cold water, the mind was simultaneously affected in the same way.

Another participant equated winter bathing with mindfulness, whereby the activity provided the participant with a clear mind and mastery of being in the present. These effects were also observed in mindfulness practices [56]. P1 also emphasized that the winter bathing club became a sanctuary, where she could always find tranquility in a hectic everyday life, which she found more convenient than a conventional mindfulness practice.

In further elaboration, a third participant explained that she also had used yoga to calm her mind, but her experience was that winter bathing was more suitable for achieving the desired outcome:

*“I also practice yoga, but sometimes the mind still races. Sometimes it can be challenging to sit and meditate and not think about anything. But when you get into the cold water, you just can’t think about anything else. It’s like the body, with the cold and warmth just forces you to be in the moment”.*

(P3)

The quote above described how the participant could achieve mental calmness and be present in the moment by using the body. According to the perspectives of Merleau-Ponty [34,45], perception and sensing are equivalent to bodily existence in the world. Thus, the body was both in contact with the world and consciousness, as the quote above illustrated. When the body was exposed to a significant number of cold stimuli, the individual automatically could do nothing else than being present in the moment.

All participants described how being in the ice-cold water stopped the stream of thoughts and described how winter bathing provided mental calmness that persisted afterwards. One of the participants described feeling dependent on winter bathing, and she even experienced more stress symptoms during periods without winter bathing.

#### 3.1.6. Summing Up

The themes above provided an in-depth insight into the participants’ experiences with regular winter bathing. The significance of these themes varied among the participants, and they often overlapped. A common red thread among the participants’ experiences was that winter bathing had contributed several positive elements to their lives, providing them with resources to cope with various challenges. This analysis formed a clear picture of the bodily experiences associated with winter bathing having a positive impact on the participants’ mental health, including their comprehensibility, manageability, and meaningfulness [33]. Additionally, the participants felt that this increased sense of coherence was implemented in other situations in their daily lives. The social aspect was also considered a significant factor in strengthening the participants’ engagement, creating a space where a unique bond of trust was evoked, allowing for reflection on their own life situations. The placement of winter bathing in blue space environments fostered a sense of security and tranquility among the participants, which could be traced back to their upbringing, interpreted through Wilson’s biophilia theory that humans find a special comfort in nature [43,44].

## 4. Discussion

The aim of this study was to explore the experiences from healthy young adults doing regular winter bathing outdoors in blue nature and how this could affect mental health. Furthermore, the aim was to create relevant knowledge for health professionals to meet the need for novel approaches to increase mental health and promote general health.

Our main findings were that participating in winter bathing regularly 1–3 times a week for a minimum of one year and up to three years had a positive impact on the participants’ mental health and well-being. Participants experienced winter bathing comprehensibly, manageably, and meaningfully. They felt that improved mental peace and a sense of coherence were transferred to other situations in their daily lives and to life in general. The fact that a relatively short exposure (seconds–minutes) to ice-cold water provided mental peace that carried over to the rest of the day and daily life in general suggests the strong potential for winter bathing to promote mental health. Emphasized qualities were having a break from a hectic everyday life and being immersed in nature, and the sense of connection with oneself, others, and nature. Winter bathing simultaneously created peace in the participants’ minds and through strong ice-cold sensory stimuli on the body, prevented thinking of anything else and forced them to be present in the moment. It is interesting that the relatively short exposure (seconds–minutes) to ice-cold water provided so much mental peace. Encountering the natural diversity of bodies contributed to a more relaxed acceptance in relating to one’s own body and to oneself. Blue nature and the placement of winter bathing in blue space environments brought about positive emotions and inner calm, as well as memories from and connection to one’s childhood and upbringing as a basis for feeling safe and secure. Facilitated by the club environments, winter bathing also served as a space for community building in which meaningful social interactions and connections occurred, and a space where a unique bond of trust was evoked, allowing for reflection on their own life situations. The social aspect was also considered a significant factor in strengthening the participants’ engagement and motivation. Winter bathing contributed to a feeling of doing something healthy for ‘one’s body and mind—for oneself’. Taking part in winter bathing was driven by a sheer inner pleasure, as the activity was enjoyable in itself. Altogether, a strong intrinsic motivation for continuing winter bathing in the club was created and this constituted a basis for persistent increased mental health and health promotion. Indicators of long-term impacts were the transfer of mental peace to everyday life together with a feeling of more resilience, meaning, and mastering in life.

Our findings are important because they meet with the need for research, especially qualitative studies, to gain deeper insight into people’s experiences with winter bathing, and preferably, experiences of long-term impacts for more than one year [24].

Winter bathing provided both physical and mental benefits for the participants, including ways to achieve rest, peace, and energy, giving the brain a break, and finding a place free of noise from the surrounding world. The question is why? Were these benefits caused by physiological changes or through altered ways of relating and dealing with life’s challenges?

All the participants reported having a routine that focused on their breathing. They used their breath to endure the ice-cold water, which helped them manage and withstand the challenges of being in such cold conditions. Winter bathing turned out to be more than a cold shock in ice-cold water; it contributed to greater resilience, added meaning to their lives, and improved their ability to handle life’s challenges.

The theories of Antonovsky about salutogenesis [33] and the development of a sense of coherence [33,57] may help us to understand why and how the experiences from winter bathing contributed to managing life better in general. The three important factors for being able to manage the experiences and challenges through life, for having a sense of coherence, and for developing and maintaining well-being are comprehensibility, manageability, and meaningfulness [33]. Comprehensibility is primarily the cognitive aspect about understanding the challenges through life, and when experienced predictably and with some structure, comprehensibility increases [33]. This refers to when an individual experiences the ice-cold water and comprehends a bodily reaction to it. Manageability is the behavioral aspect about the individual’s resources, whether inner resources and/or support from others, and the ability to manage challenges through life. Strong manageability enables the individual to manage many challenges, to cope with and solve problems, and to see them as challenges rather than burdens. It can be developed through activities that are not too easy and not too difficult [33]. In this case, the winter bathers began their travels in the summertime and increased their capacity through the season. Meaningfulness is the most important and most motivational aspect about feeling and understanding the meaning of investing energy and going through the challenges and with that, it is very much about the motivation for going through and enduring the challenges of life and feeling the meaning of life [33]. Much of this was developed through participation in society, in this case, the winter bathing club. According to these theories [33], the participants were able to comprehend, to manage, and to acknowledge the meaning of life by enduring the ice-cold water and by that, they obtained more resilience and meaning in life and mastered life better.

Through winter bathing, the participants experienced a break for the busy mind, peace in the mind, and more presence in the moment, all of which are supported by Merleau-Ponty’s perspectives on body and mind being inseparable and the body being an anchor to the present moment [34,45,46]. Being in the ice-cold water prevented thinking of anything else, as if the body and the coldness helped one to be in the present moment. The heat in the sauna had a similar effect. The body, the ice-coldness, and the heat made it possible to be fully in the present moment, free from thinking about the past and the future. The experienced break from rumination is emphasized by the understanding of the body being an anchor to the present [34], with the body being the closest we can come to the present [45], and understanding that the body is the place for the present moment and for being fully in the present moment [58].

The experienced break from rumination was also found in another study investigating stand-up paddling’s impact on mental health [59]. In this study, the break from rumination was not caused by the cold water, but by the full concentration of balancing on an SUP board. A follow-up study revealed long-term impacts on rumination and mental health from SUP activity [60].

Different therapies such as body awareness therapy, mindfulness, and yoga also focus on awareness of the present moment [56,61,62,63]. Some participants in our study noted that winter bathing, like mindfulness, may contribute to coping competencies by helping participants to stay present and to maintain a clear mind. A participant emphasized that the winter bathing club had become a sanctuary where she always found peace in a hectic daily life, and another participant underlined that she also used yoga to achieve mental peace, but she experienced winter bathing being much better for this purpose. Our results are supported by a systematic review of quantitative studies from 2017 [64] showing that blue nature had a positive impact on mental health and well-being. In both studies, the participants achieved peace and experienced more energy by staying in blue nature.

Besides mental peace and a break for the busy mind, the participants also emphasized that winter bathing in the club contributed to social connectedness. The participants experienced connectedness and a relationship of trust to the club and the club members, and we were surprised how much the participants emphasized the importance of this social aspect while it apparently is an individual activity. According to the self-determination theory by Deci and Ryan [38,39,41], the experienced relatedness and connectedness could have contributed to the above-mentioned strong intrinsic motivation for participating in winter bathing.

At the same time, blue nature appeared to be instrumental and deeply integrated into winter bathing and helped the participants to settle down. It is interesting that the relatively short exposure (seconds–minutes) to ice-cold water provided so much mental peace.

Our results emphasized the importance of winter bathing’s many social aspects, especially regarding motivation. These findings are supported by a qualitative study from 2023 by Flynn et al. [65] concluding that the winter bathing club constituted an inclusive milieu for both new and experienced winter bathers, who preferred to bathe in a club because of the social interactions and connectedness, besides safety-related reasons. This study also supports our findings on winter bathing’s positive and strengthening impact on mental health, helping participants achieve mental peace and reduced stress from the ice-cold shock [65].

Another cross-sectional study from 2021 by Demori et al. exploring the effects of winter sea bathing on psycho-neuro-endocrine-immunological parameters [66] also supports our results with an increased sense of coherence when taking part in winter bathing. This study indicates that when driven from the ice-cold shock, winter bathers have an increased ability to turn negative stress into positive stress, leading to increased well-being and mental health.

It is interesting that the relatively short exposure (seconds–minutes) to ice-cold water provided so much mental peace. On a biochemical level, science can help to shed light on some of the mechanisms behind the immediate mood lift from the ice-cold shock which is explained by an instant release of neurotransmitters that play a crucial role in emotional regulation, stress regulation, and reward processing [67]. However, the mental peace that the participants in our study experienced sometimes lasted throughout the day, and was also transferred to other life situations. This is worthy of further investigation in order to better understand the underlying mechanisms, both psychological and physiological, and to explore the possible long-term effects.

The significant impact of only a short dip in cold water is supported by a study showing that the therapeutic benefits of cold-water immersion can be achieved from a single exposure [68]. Yet, there is a great variation in studies exploring cold-water immersion in relation to mental health and there is a need to examine the relationship between, e.g., water temperature, time spent in the water, and number of sessions in order to provide a more targeted approach to cold-water bathing for health promotion. Future research is necessary to fully understand these health benefits.

A review by Espeland et al. from 2022 [1] emphasized the beneficial health effects of exposure to cold water, and the review supports our results about the participants’ experiences of mastering the activity of enduring being in the cold water, of building up competencies to manage to regulate heat in the body, and of experiencing less discomfort when exposed to coldness.

The salutogenetic theories by Antonovsky [33] support our results of the experienced factors providing health rather than disease. All participants in our study felt well and healthy, giving us a unique opportunity to focus on what is basic for health. As pointed out in the introduction section, mental healthcare costs in Denmark are enormous [7,8], and if mental and physical health decrease further, they can potentially constitute a socioeconomic burden in the future unless we work on novel approaches to increase health promotion [4,5].

Winter bathing constitutes a health-promoting low-cost initiative available for a wide age group. Regularly winter bathing for a period of a minimum of one year was associated with mental health benefits. The perspectives of Merleau-Ponty concerning body and mind as a whole and inseparable [34,45,46,69] support that physical influence on the body causes mental health improvements. The physical body influences the mind and the mind influences the body.

During the COVID-19 pandemic, an increase in the use of nature during an unsafe period proved to be of great importance [70], and in recent years, an increasing focus on including the surrounding nature in physiotherapeutic and health professional practice and rehabilitation has arisen [71]. As an example, a study on a project called SPARK (Sound, Park, Activities, Rehabilitation, and Climate) was carried out in 2019–2021 in Denmark, where rehabilitation services are provided free of charge as part of the legislated Danish welfare system [72]. A 7.2-hectare urban green space was transformed into an innovative outdoor living rehabilitation nature reserve in the city center of Aarhus, Denmark, also supporting climate adaptations as well as health promotion, and encouraging people to engage in physical and social experiences. Nature helped the participants recall earlier relations and experiences and establish newfound meanings and uses of the outdoors in response to a changed life situation, and it appeared to enhance the agency of people with disabilities. It appeared that outdoor nature experiences gradually built autonomy inside and outside of the rehabilitation context and the participants experienced taking an increased active part in their own lives. They also emphasized a feeling of connectedness to their surroundings and of being a part of a strong community [72].

Likewise, in our study, we found that winter bathing fostered an increased sense of coherence and a feeling of a connectedness. Participants experienced a connectedness to nature as well as social connectedness and a strong social relationship to the members of the winter bathing club, which strengthened their motivation for the activity as a meaningful part of their lives. All this seemed to be transferred to their daily lives. Our study also found that nature could bring about memories from and connection to one’s childhood and upbringing as a basis for feeling secure, interpreted through the biophilia theory that humans find a special comfort in nature.

According to the assumption based on the theory of Merleau-Ponty [34]—that ‘I am my body’, that body and mind are ‘one thing’, that there are no two things to separate, they cannot be separated from each other—body and mind will influence each other and in other words, the effect can yield either result. According to this, the experiences most likely have influenced the participants’ physical health too, and in this perspective, the participants’ experiences presumably not only influenced their mental health but more likely their health in general.

### 4.1. Summing Up

The participants in this study experienced increased peace, more energy, and in general, increased mental health through winter bathing. Several studies, both qualitative and quantitative, support these results. Other studies support our results about winter bathing also having a positive influence on the participants’ social connectedness which strengthened their motivation. Nonetheless, research in blue nature and winter bathing is still relatively new and thereby sparse, which emphasized the need for further research. Winter bathing hopefully inspires novel and alternative approaches to motivate and promote people’s health.

### 4.2. Limitations and Strengths

This case study consists of only four participants, which limits the generalizability and calls attention to the need for more research exploring the experiences and long-term influences of winter bathing. Nevertheless, our findings are important because they meet the need for research, especially qualitative studies, to gain deeper insight into people’s experiences with winter bathing, including long-term experiences and impacts for more than one year [24].

The age of the participants, 25–32, weakens the transferability to a broader population. On the contrary, it strengthens the transferability to the specific group aged 25–32, which is exactly one of the two groups with the largest decrease in mental health from 2010 to 2023 [3] and therefore is of great relevance.

This study mainly focused on the participants’ experiences with winter bathing in the present time. We could have explored the participants’ memories about their baseline of mental health more, although it would have depended on their memories, causing some potential memory bias. We did not have the opportunity to interview the participants before they started winter bathing. To further clarify the picture of the effects of winter bathing, a cohort study that interviews the participants before they start winter bathing will strengthen the results.

The validity could have been strengthened more by fully participating in the cold water and in the sauna, which we recommend for further research. On the contrary, by moving our fieldwork and interviews outside in blue nature near the sea, walking and sitting together, the participants felt comfortable and secure in their well-known surroundings, the atmosphere became informal, and the participants found it easy to recall experiences, feelings, thoughts, etc., which strengthened the validity of this study.

The fact that all authors had lived close to blue nature and were familiar with the context of participating in winter bathing and also had positive experiences in this area made each author a sort of ‘native’ anthropologist, and it is sometimes questioned if it is then possible to create a necessary distance to the participants and the field, but on the other hand, it is also assumed that the native anthropologist is able to contribute with more authentic knowledge than the non-native anthropologist [49]. We experienced that having bodily experiences ourselves led to an embodied understanding of the participants’ lives and world which made the interviews more empathic. The shared experiences of participating in winter bathing functioned as a unique basis for elaborating meaningful questions and dialogue, and the participants were very open minded in telling us about their experiences.

Our preunderstanding according to being physiotherapists probably influenced the research questions and thereby the participants’ answers in a direction focusing on bodily experience direction rather than, for example, on medication or nutrition. According to the aim of this study, exploring experiences and bodily experiences on mental health, we consider that the influence in this case has been appropriate.

## 5. Conclusions

In conclusion, regularly winter bathing 1–3 times a week, for a minimum of one year and up to three years, had a positive impact on the participants’ mental health and well-being, including their comprehensibility, manageability, and meaningfulness, and the participants felt that mental peace and an increased sense of coherence were transferred to other situations in their daily lives and to life in general. The fact that a relatively short exposure (seconds–minutes) to ice-cold water provided mental peace transferred to the rest of the day and to daily life in general suggests the effectiveness of winter bathing. Emphasized qualities were having a break from a hectic everyday life and being immersed in nature, and the sense of connection with oneself, others, and nature. Winter bathing simultaneously created peace in the participants’ minds, and through strong ice-cold sensory stimuli on the body, prevented thinking of anything else and helped them to be present in the moment. Encountering the natural diversity of bodies contributed to a more relaxed and accepting relationship with one’s own body and self. Blue nature and the placement of winter bathing in blue space environments brought about positive emotions and inner calm, as well as memories from and connection to one’s childhood and upbringing as a basis for feeling safe and secure. Facilitated by the club environments, winter bathing also served as a space for community building in which meaningful social interactions and connections occurred, a space where a unique bond of trust was evoked, allowing for reflection on their own life situations. The social aspect was also considered a significant factor in strengthening the participants’ engagement and motivation. Winter bathing contributed to a feeling of doing something healthy for ‘one’s body and mind—for oneself’. Winter bathing was driven by a sheer inner pleasure, as the activity was enjoyable in itself, and altogether, a strong intrinsic motivation for continuing winter bathing in the club was created and established and therefore was also a possible basis for more persistent increased mental health and health promotion in the long run. Indicators of long-term impacts were the transfer of mental peace to everyday life together with greater resilience, added meaning to life, and improved ability to handle life’s challenges.

Our findings are important because they meet the need for research, especially qualitative studies, to gain deeper insight into people’s experiences with winter bathing and preferably experiences of long-term impacts for more than one year [24].

## 6. Perspectives

Winter bathing can exemplify a return to the body in a digitalized world—a world with visual inputs from an iPhone, iPad, computer, etc. Sensing the world through our eyes has increased and this may be at the expense of sensing the world with our other senses such as, for example, a sense of touch, e.g., sensing the water all around one’s body; a sense of smell, e.g., smelling the water; a sense of hearing, e.g., sensing the sound of the waves.

For some people, winter bathing can constitute a health-promoting low-cost and relatively less time-consuming initiative available for a wide age group, and countries with lakes, seas, and rivers have many possibilities for this. The fact that a relatively short exposure (seconds–minutes) to ice-cold water provided mental peace transferred to the rest of the day and to daily life in general suggests the potential effectiveness of winter bathing for promoting mental health. We all are motivated by different things, and we respond differently. Not everyone has experiences with and preferences for participating in mindfulness or exercising, e.g., in a fitness center or with exercising at all, as Strömberg found in a study where people with chronic obstructive pulmonary disease described exercise training as something unknown and unimportant for them [73]. However, ‘it’ does not need to take place in a gym or in a fitness center; the important thing is finding and doing something meaningful, and blue nature and winter bathing hopefully can inspire novel approaches supplementing traditional rehabilitation activities and meeting the needs and wishes of people who do not feel motivated or comfortable in a traditional training room. Rehabilitation can be supplemented—or combined—with activities in blue nature, and winter bathing appears to hold a great potential for health promotion. This study suggests supplementary alternatives to training and fitness rooms. A close connection and connectedness between blue nature, bodily experiences, and participation in a unique social community contributed to the positive impact on the participants’ mental health. This has great implications for health professionals as a basis for creating alternative and innovative initiatives for motivating people to take care of their own health, appreciating that we are all different [74]. Furthermore, by staying in nature and experiencing a connection to it and a sense of being part of it, people may develop a greater propensity to take better care of nature [75]. This could catalyze more environmentally conscious behaviors, which are essential for addressing today’s climate and ecological challenges, ultimately promoting more sustainable practices.

## Data Availability

The original contributions presented in this study are included in this article; further inquiries can be directed to the corresponding author.

## Appendix A

**Table A1:** Interview guide for participants.

Briefing		
Presentation of the interviewers and the aim/purpose of the project	Who are we?	The group consists of: Anders, Anne, Linda, and Nikolaj.
	The aim of the interview	The aim of this interview is to hear about your experiences during winter bathing and how winter bathing influences you.
	Timeframe	The interview is expected to take approximately 30 min.
The settings for the interview	Make aware that the interview will be recorded using a mobile phone	We want to inform you that the interview will be recorded on a mobile phone. The recording is used as a support for our memory and will be part of our project work.
	Anonymization	The interview will be treated and stored confidentially and will be deleted after usage. During the work on this project your statements will be anonymized, so no information can be traced back to you.
	Roles during the interview setting	My task is to ask the questions, while my fellow researcher’s role is taking notes, manage the mobile phone, and possibly ask follow-up/elaborative questions during the interview.
	Statement	Please feel free to ask if there is anything you are unsure about or do not understand during the interview. We want to make you aware that your participation in the interview is voluntary, and you can always withdraw your consent. You can choose not to answer individual questions.
Research question	Interview questions	
1. Winter bathing (mentally and physically)	Why do you engage in this activity? (preventive or promotional)	
	Mental:	
	Which thoughts go through your mind when you are winter bathing?	
	Which sensations do you experience when entering the water?	
	Which sensations do you experience when coming out of the water?	
	Do you feel a greater sense of calmness in your mind after bathing?	
	Physical:	
	Which bodily sensations do you experience when entering the water?	
	Which bodily sensations do you experience when coming out of the water?	
	Does your body feel different before and after bathing?	
	Do you feel cold when bathing? Does being in the water warm you up?	
2. Background information/nature’s role in your life	Age	
	How long have you been winter bathing?	
	How many times per week do you winter bathe?	
	How did you start?	
	Leisure interests	
	What does nature mean to you?	
	Do you engage in other activities in nature?	
	What can the ocean do for you?	
	Educational level	
	Occupation	
3. Social aspects	Your motivation behind winter bathing?	
	What prompted you to start?	
	Do you do it alone or with others?	
	Are there social events in the club?	
	What does the social aspect mean to you?	
4. What does winter bathing contribute to your life?	Your journey with winter bathing from the beginning until now.	
	Could you live without winter bathing in your life?	
	How does it influence your day afterwards?	
	Can you feel a difference on days when you do not winter bathe vs. days you do?	
	What does it do for your health? (mentally and physically)	
Debriefing		
Conclusion	The interview is near to its end.	
Practical	We will have completed the project in mid-January. If you are interested, we can provide you with the results via email.	
Summary	Thank you very much for your cooperation. Feel free to contact us if you have any questions.	
